# Effect of *Alpinia zerumbet* components on antioxidant and skin diseases-related enzymes

**DOI:** 10.1186/1472-6882-12-106

**Published:** 2012-07-24

**Authors:** Jamnian Chompoo, Atul Upadhyay, Masakazu Fukuta, Shinkichi Tawata

**Affiliations:** 1 Department of Bioscience and Biotechnology, The United Graduate School of Agricultural Science, Kagoshima University, 1-21-24 Korimoto, Kagoshima, 890-0065, Japan; 2 Department of Bioscience and Biotechnology, Faculty of Agriculture, University of the Ryukyus, Senbaru 1, Nishihara-cho, Okinawa, 903-0213, Japan

**Keywords:** *Alpinia zerumbet*, Antioxidant, Skin diseases-related enzymes, 5,6-Dehydrokawain (DK)

## Abstract

**Background:**

The skin is chronically exposed to endogenous and environmental pro-oxidant agents, leading to the harmful generation of reactive oxygen species. Antioxidant is vital substances which possess the ability to protect the body from damage cause by free radicals induce oxidative stress. *Alpinia zerumbet*, a traditionally important economic plant in Okinawa, contains several interesting bioactive constituents and possesses health promoting properties. In this regard, we carried out to test the inhibitory effect of crude extracts and isolated compounds from *A. zerumbet* on antioxidant and skin diseases-related enzymes.

**Methods:**

The antioxidant activities were examined by DPPH, ABTS and PMS-NADH radical scavenging. Collagenase, elastase, hyaluronidase and tyrosinase were designed for enzymatic activities to investigate the inhibitory properties of test samples using a continuous spectrophotometric assay. The inhibitory capacity of test samples was presented at half maximal inhibitory concentration (IC_50_).

**Results:**

The results showed that aqueous extract of the rhizome was found to have greater inhibitory effects than the others on both of antioxidant and skin diseases-related enzymes. Furthermore, 5,6-dehydrokawain (DK), dihydro-5,6-dehydrokawain (DDK) and 8(17),12-labdadiene-15,16-dial (labdadiene), isolated from rhizome, were tested for antioxidant and enzyme inhibitions. We found that DK showed higher inhibitory activities on DPPH, ABTS and PMS-NADH scavenging (IC_50_ = 122.14 ± 1.40, 110.08 ± 3.34 and 127.78 ± 4.75 μg/ml, respectively). It also had stronger inhibitory activities against collagenase, elastase, hyaluronidase and tyrosinase (IC_50_ = 24.93 ± 0.97, 19.41 ± 0.61, 19.48 ± 0.24 and 76.67 ± 0.50 μg/ml, respectively) than DDK and labdadiene.

**Conclusion:**

Our results indicate that the rhizome aqueous extract proved to be the source of bioactive compounds against enzymes responsible for causing skin diseases. Moreover, DK could be used as a potent inhibitor and be further exploited to be used in anti-skin disease formulations.

## Background

The skin is the largest organ of the human, both in terms of surface area and weight, which serves as an important environmental interface providing a protective envelope that is crucial for homeostasis. On the other hand, the skin is a major target for toxic insult by a broad spectrum of physical (UV radiation) and chemical (xenobiotic) agents that are capable of altering its structure and function [1]. Many environmental pollutants are oxidants or catalyze the production of reactive oxygen species (ROS) directly or indirectly. ROS act as cell-signaling molecules and can react with DNA, proteins, fatty acids and saccharides causing oxidative damages. Such injuries result in a number of harmful effects (disturbed cell metabolism, morphological and ultrastructural changes), attack on the regulation pathways, and alterations in the differentiation, proliferation and apoptosis of skin cells [2]. The skin possesses an array of defense mechanisms that interact with toxicants to obviate their deleterious effects. These include non-enzymatic and enzymatic molecules that function as potent antioxidant or oxidant-degrading systems [3]. For screening antioxidants, the methods based on a single relatively stable reagent such as DPPH^·^ and ABTS^·+^ have most popular, because of their simple set-up and ease of control [4].

Collagenase is one of the few proteinases capable of degrading the triple-helical region of native collagen under a physiological condition. Collagen is the fibrous component of the extracellular matrix (EMC) in the skin and the collagen content in the skin decreases greatly during the aging process and due to long-term exposure to UV radiation. Inhibition of collagenase plays an important role in protecting unbalanced turn over or rapid breakdown of collagen in human inflamed or UV-irradiated skin [5]. Elastase is a proteolytic enzyme involved in the degradation of the EMC that includes elastin. Loss of elastin is a major part of what cause visible signs of aging (wrinkles, sagging) in the skin. Elastase secretion and activation caused by exposure to UV light or ROS, an approach that inhibits the elastase activity, could also be applied as a useful method to protect against skin diseases [6]. Hyaluronidase is a mucopolysaccharase that hydrolyzes glycosaminoglycans, including hyaluronic acid, in the EMC during tissue remodeling. When the level of hyaluronic acid decreased under conditions in which the hyaluronidase activity is increased, the moisture and tension of the skin are reduced. Thus, hyaluronidase inhibitors are useful cosmeceutical ingredients as they have anti-wrinkle and anti-aging effects on the skin [7]. Tyrosinase is known to be a key enzyme for the melanin biosynthesis, the compound largely responsible for hair and skin color in mammals. Melanin pigments play a crucial protective role against skin photocarcinogenesis. Tyrosinase inhibitors may be clinically helpful in dealing with skin diseases [8].

*Alpinia zerumbet* (Family Zingiberaceae) is a medicinal plant found in several islands of Japan, including Okinawa. Several pharmacological effects of essential oil from the leaves of *A. zerumbet* have been used for skin care, insect repellent and deodorant products [9], antinociceptive effects on mice [10] and antihypertensive and cardiovascular effects on rat [11]. We have reported phenolic compounds and their antioxidant activities in leaves and rhizomes of *A. zerumbet*[12] and have isolated active compounds from the rhizomes of *A. zerumbet* against HIV-1 integrase and neuraminidase enzymes [13]. Recently, we have reported the inhibitory effects of this plant on advanced glycation end products formation [14]. Considering all these aspects, the present study was undertaken to evaluate antioxidant and anti-enzymatic activities of *A. zerumbet* for prevention of the skin diseases.

## Methods

### Chemicals

1,1-Diphenyl-2-picrylhydrazyl (DPPH), *tert*-butyl hydroxytoluene (BHT), kojic acid, gallic acid, 2,2´-azino-bis(3-ethylbenzothiazoline-6-sulfonic acid)diammonium salt (ABTS), nitro blue tetrazolium (NBT), phenazine methosulfate (PMS), bovine serum albumin (BSA) and Folin-Ciocalteu reagent were purchased from Wako Pure Chemical Industries, Ltd (Japan). Nicotinamide adenine dinucleotide-reduced (NADH), N*-*succ-(Ala)3-nitroanilide (SANA), *N-*[3-(2-furyl)acryloyl]-Leu-Gly-Pro-Ala (FALGPA), collagenase, elastase, hyaluronidase, tyrosinase, hyaluronic acid and oleanolic acid were secured from Sigma-Aldrich, Inc. (USA).

### Preparation of plant extracts

The six structures (rhizomes, stems, leaves, flowers, pericarps, and seeds) of *A. zerumbet* were collected at the University of the Ryukyus, Okinawa, Japan. Aqueous extract was obtained by boiling 10 g of air-dried sample for 20 min. For ethanol extract, samples were immersed in ethanol for 24 h. The aqueous and ethanol extracts were filtered, dried under vacuum and then dissolved in corresponding solvent (water and methanol, respectively) for further analysis.

### Isolation and quantification of DK, DDK and labdadiene

The isolation of DK and DDK were isolated from rhizomes of *A. zerumbet* as reported previously [13]. DK and DDK were purified using a TSK gel ODS-100Z column (15 x 0.46 cm i.d.; 5 μm particle size) (Tosoh Corp, Japan) and monitored continuously at 280 nm. The mobile phase consisted of water with 0.1% acetic acid (solvent A) and methanol with 0.1% acetic acid (solvent B) at a flow rate of 0.8 ml/min. The gradient elution was performed as follows: 1–10 min, 50% B isocratic, 10–20 min, linear gradient 80-100% B, and 20–30 min, 100% B [14].

Labdadiene was isolated by reported previously [14] and was collected at 235 nm using TSK gel ODS-100Z column. The mobile phase was water with 0.1% acetic acid (solvent A) and methanol with 0.1% acetic acid (solvent B) at flow rate of 0.8 ml/min. The gradient elution was performed as follows: 0–10 min, 80% B isocratic; 10–20 min, linear gradient 80-100% B, 20–40 min, 100% B isocratic.

**Figure 1:**
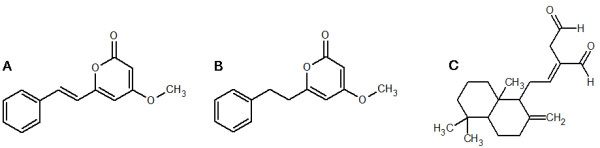
Chemical structures of 5,6-dehydrokawain; DK (A), dihydro-5,6-dehydrokawain; DDK (B) and 8(17),12- labdadiene-15,16-dial; labdadiene (C).

The quantification of each compound in six different structures of *A. zerumbet* with aqueous and ethanol extracts was determined based on peak area measurement by the same processes as described above. The structures of DK, DDK and labdadiene are shown in Figure 1. The isolated compounds were dissolved methanol for further analysis.

### Antioxidant assays

#### DPPH radical scavenging activity assay

The free radical scavenging activity was evaluated as described previously [15]. Five hundred microlilters of the different concentration of test samples were mixed with 200 μl of 0.5 mM DPPH methanol solution and 500 μl of 0.1 M sodium acetate buffer (pH 5.5). After shaking, the mixture was incubated in the dark at room temperature for 30 min, and then the absorbance was measured at 517 nm. BHT was used as a positive control, while water and methanol were used as controls for calculation.

### Total antioxidant activity (ABTS) assay

The total antioxidant activity of test sample was measured with modified method as described previously [16]. ABTS^·+^ solution was generated by mixing 7 mM ABTS and 2.45 mM potassium persulfate (K_2_S_2_O_8_) in water, which was placed in the dark at room temperature for 16 h to give the complete oxidation of ABTS. Before using, the ABTS^·+^ solution was diluted with water to get an absorbance of 0.700 ± 0.050 at 734 nm. Briefly, 1 ml of ABTS^·+^ solution was added to 30 μl of test samples and mixed thoroughly. BHT was used as positive control and the sample solutions were used as controls. The reactive mixture was incubated at room temperature for 6 min and the absorbance was immediately recorded at 734 nm.

### PMS-NADH system superoxide-radical scavenging assay

The superoxide scavenging activity was assayed following the method of Lau et al. [17] with minor modifications. The reaction mixture contained 0.5 ml of 105 μM NADH, 0.5 ml of 66 μM NBT dissolved in 0.1 M phosphate buffer (pH 7.4) and 0.1 ml sample in different concentrations. The reaction was initiated by adding 0.5 ml of 30 μM PMS into the reaction mixture. Methanol and water were used as a control and BHT as a positive control. After 10 min, the reaction mixture reached to stable color, measurement of absorbance was conducted at 560 nm.

The ability of the extracts to scavenge DPPH, ABTS^·+^, PMS-NADH radicals were calculated by following formula:

Radical scavenging (%) = [(O.D_control_ – O.D_sample_)/ O.D_control_] x 100,

where O.D is the optical density in the presence or absence of the samples.

### Determination of total phenolic contents

The amount of total phenolics was determined using the Folin-Ciocalteu reagent method [18]. Briefly, 0.5 ml of Folin-Ciocalteu reagent and 0.5 ml of distilled water were added to 0.2 ml of each extract dissolved in corresponding solvent (1 mg/ml). After 1 min, 0.8 ml of sodium carbonate solution (7.5%) was added in mixture and incubated at room temperature for 30 min. Absorbance was measured at 760 nm. Total phenolic contents were expressed as gallic acid equivalents (GAE) in milligrams per gram extract.

### Enzymatic assays

#### Collagenase inhibition assay

Collagenase inhibition assay was performed by the method described previously [19] which based on the hydrolysis of *N-*[3-(2-furyl)acryloyl]-Leu-Gly-Pro-Ala (FALGPA) by collagenase to produce FA-Leu and Gly-Pro-Ala. The assay was performed in 50 mM Tricine buffer (400 mM NaCl and 10 mM CaCl_2_, pH 7.5). Collagenase from *Clostridium histolyticum* (ChC) was dissolved in the buffer for use at an initial concentration of 0.8 units/ ml. The synthetic substrate, FALGPA, was dissolved in the Tricine buffer to 2 mM. Sample extracts were incubated with the enzyme in the buffer for 15 min before adding substrate to start reaction. The final reaction mixture (150 μl total volume) contained Tricine buffer, 0.8 mM of FALGPA, 0.1 units of ChC and 25 μg of test extracts. Controls were performed with water and methanol, while oleanolic acid as a positive control. After adding substrate for 20 min, collagenase activity was measured at 340 nm.

### Elastase inhibition assay

Elastase inhibition was assayed using *N-*succ-(Ala)3-nitroanilide (SANA) as the substrate, monitoring the release of *p*-nitroaniline by the method described [20] with a few modifications. The inhibitory activity determined the intensity of color released during cleavage of SANA by the action of elastase. Briefly, 1 mM SANA was prepared in 0.1 M Tris–HCl buffer (pH 8.0) and this solution (200 μl) was added to the stock sample solution (20 μl). The solutions were vortexed and preincubated for 10 min at 25 °C and then 20 μl of elastase from porcine pancreas (0.03 units/ml) was added. After vortexing, each solution was placed in a water bath at 25 °C for 10 min and the absorbance was measured at 410 nm. Controls were performed with water and methanol, while oleanolic acid as a positive control.

### Hyaluronidase inhibition assay

Hyaluronidase inhibitory assay was performed by the method described previously [21] which depends on the fact that substance forms a precipitate with protein in an acidic solution. A test sample of 5 μl was pre-incubated with hyaluronidase from bovine test (1.50 units in 100 μl), sodium phosphate buffer 20 mM (pH 7.0) with sodium chloride 77 mM and bovine serum albumin (BSA) 0.01% for 10 min at 37 °C. Subsequently, the assay was initiated by adding hyaluronic acid sodium salt from rooter comb 100 μl (0.03% in 300 mM sodium phosphate, pH 5.35) to the incubation mixture and incubated further for 45 min at 37 °C. Hyaluronic acid (undigested) was precipitated with acid albumin solution (1 ml), made up of bovine serum albumin 0.1% in sodium acetate 24 mM and acetic acid 79 mM (pH 3.75). It was allowed to stand at room temperature for 10 min and then the absorbance was measured at 600 nm. Oleanolic acic was used as positive control and the sample solutions were used as controls.

The percentage inhibition for collagenase, elastase and hyaluronidase assays were calculated by:

Enzyme inhibition activity (%) = (1– B/A) x 100, where A is the enzyme activity without sample and B is the activity in presence of the sample.

### Tyrosinase inhibition assay

Tyrosinase activity inhibition was determined by the method as described previously [22] by measuring the DOPA chrome formed due to the action of tyrosinase enzyme on tyrosine substrate. In brief, sample extracts were dissolved in corresponding solvent to make the different concentrations (μg/ml). The 96-well plate was set up in the following order; 120 μl of phosphate buffer (20 mM, pH 6.8), 20 μl of sample and 20 μl of mushroom tyrosinase (500 units/ml in 20 mM phosphate buffer). After incubation at 25 °C for 15 min, reaction was initiated by adding 20 μl of 0.85 mM L-tyrosine solution to each well. The enzyme activity was determined by measuring the absorbance at 470 nm. Kojic acid was used as a positive control. The percentage of tyrosinase inhibition was calculated as follows:

Tyrosinase inhihition (%) = [(A – B) – (C – D)]/ (A – B) x 100, where A is the absorbance of the control with the enzyme, B is the absorbance of the control without the enzyme, C is the absorbance of the test sample with the enzyme and D is the absorbance of the test sample without the enzyme.

### Statistical analysis

The data were analyzed by one-way ANOVA using SPSS version 16.0 for Windows. Upon significant difference, means were separated using Tukey HSD range test at *p* = 0.01 with three replications. In some cases, only means and standard deviation of the sample means are presented.

## Results

The radical scavenging activities of aqueous extracts were found superior to ethanol extracts (*p* = 0.01). The seed and rhizome had better antioxidant activities. For DPPH assay, seed extracts showed the best activity (IC_50_ = 10.33 ± 0.03 μg/ml) followed by rhizome (IC_50_ = 25.31 ± 0.67 μg/ml). In case of ABTS radical scavenging activity, the IC_50_ values for rhizome and seed extracts were 73.94 ± 1.23 and 84.29 ± 0.72 μg/ml, respectively. The scavenging of superoxide radicals also showed both seed and rhizome extract to have better activities than other parts with IC_50_ of 58.55 ± 0.31 and 64.70 ± 0.72 μg/ml, respectively. However, rhizome and seed aqueous extracts had weaker inhibitory effect than positive control BHT on DPPH, ABTS and PMS-NADH radical scavenging assays (IC_50_ = 11.74 ± 1.23, 14.26 ± 0.16 and 24.80 ± 0.98 μg/ml) (*p* = 0.01) [Table 1]. It was also found that the seed and rhizome aqueous extracts contained higher amounts of phenolic compounds (187.75 ± 0.97 and 124.51 ± 0.57 GAE mg/g extract, respectively) [Table 2].

**Table 1:** **Radical scavenging activity of six parts of*A. zerumbet*extracts on DPPH, ABTS and PMS-NADH superoxide**

**Sample**	**Extraction**	**Inhibitory effect (IC**_**50**_**; μg/mL)**		
		**DPPH**	**ABTS**	**PMS-NADH**
BHT		11.74 ± 0.15 a ^*1*^	14.26 ± 0.16 a	24.80 ± 0.98 a
Rhizome	Aqueous	25.31 ± 0.67 b	73.94 ± 1.23 b	64.70 ± 0.72 c
	Ethanol	145.07 ± 1.68 e	155.86 ± 4.09 f	113.37 ± 1.24 d
Stem	Aqueous	144.03 ± 0.27 e	127.97 ± 1.70 d	215.09 ± 0.28 h
	Ethanol	293.08 ± 0.17 g	223.17 ± 0.26 i	132.27 ± 1.33 e
Leaf	Aqueous	165.60 ± 0.44 f	143.77 ± 1.37 e	191.24 ± 0.20 g
	Ethanol	586.47 ± 0.77 j	206.65 ± 0.85 h	168.23 ± 4.77 f
Flower	Aqueous	132.19 ± 1.02 c	140.50 ± 1.18 e	117.32 ± 1.46 d
	Ethanol	559.01 ± 3.61 h	293.90 ± 3.64 k	210.50 ± 4.03 h
Pericarp	Aqueous	140.91 ± 0.30 de	163.41 ± 1.07 g	169.02 ± 1.01 f
	Ethanol	580.38 ± 0.26 i	246.71 ± 0.85 j	166.11 ± 0.52 f
Seed	Aqueous	10.33 ± 0.03 a	84.29 ± 0.72 c	58.55 ± 0.31 b
	Ethanol	136.63 ± 0.33 cd	137.92 ± 1.74 e	56.12 ± 1.08 b

^*1*^ The data represent the means ± SD of three determinations. Values with the same letter are not significantly different (*p* = 0.01) from each other.

**Table 2:** **Total phenolic content from six parts of*A. zerumbet*extracts**

**Sample**	**Total phenolic content (GAE mg/g extract)**	
	**Aqueous extract**	**Ethanol extract**
Rhizome	124.51 ± 0.57 b ^*1*^	38.50 ± 0.47 h
Stem	59.41 ± 0.87 d	9.60 ± 0.04 j
Leaf	47.80 ± 0.73 f	42.14 ± 0.22 g
Flower	75.15 ± 0.23 c	12.90 ± 0.11 i
Pericarp	58.42 ± 0.18 d	10.26 ± 0.04 j
Seed	187.75 ± 0.97 a	56.31 ± 0.09 e

^*1*^ The data represent the means ± SD of three determinations. Values with the same letter are not significantly different (*p* = 0.01) from each other.

**Figure 2:**
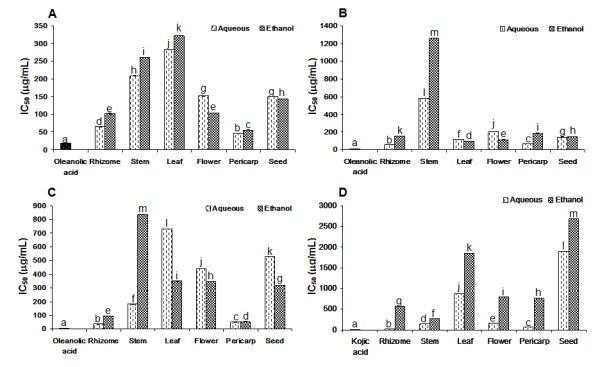
**Inhibitory effects of six parts of*A. zerumbet*on collagenase (A), elastase (B), hyaluronidase (C) and tyrosinase (D) activities.**

The effect of six different structures of *A. zerumbet* on elastase, hyaluronidase and tyrosinase inhibitions showed that rhizome had stronger inhibitory activity than the other parts (IC_50_ = 57.43 ± 0.18, 35.02 ± 0.75 and 21.84 ± 0.77 μg/ml, respectively). Pericarp showed higher inhibitory activities against collagenase (IC_50_ = 45.67 ± 0.50 μg/ml). However, test samples had weaker inhibition than positive control, oleanolic acid (IC_50_ = 17.95 ± 0.23, 10.91 ± 0.03 and 5.28 ± 0.19 μg/ml, respectively) on collagenase, elastase, hyaluronidase activities and lower ihhibitory effect than kojic acid (IC_50_ = 8.90 ± 0.22 μg/ml) on tyrosinase acitivity (*p* = 0.01) [Figure 2].

The amount of DK, DDK and labdadiene in the crude extracts of six different structures from *A. zerumbet* with aqueous and ethanol solvents extraction are presented in Table 3. DK and DDK were found in all structures of plant and in both solvent extractions. The rhizome aqueous extract contained higher amount of DK (1.23 mg/g crude extract) followed by pericarp aqueous extract and rhizome ethanol extract (1.15 and 1.14 mg/g crude extract, respectively). DDK was found in high amount in pericarp aqueous extract (0.85 mg/g crude extract) followed by rhizome aqueous extract (0.62 mg/g crude extract). Labdadiene was present in higher amount in seed ethanol and aqueous extracts (1.00 and 0.96 mg/g crude extract, respectively) followed by rhizome and pericarp aqueous extracts (0.81 and 0.75 mg/g crude extract), moreover, the crude extracts of stem, leaf and flower did not contain labdadiene.

**Table 3:** **Amount of DK, DDK and labdadiene in aqueous and ethanol extracts from six different structures of*A. zerumbet***

**Sample**	**Extraction**	**Amount of compound (mg/ g crude extracts)**		
		**DK**	**DDK**	**Labdadiene**
Rhizome	Aqueous	1.23 ± 0.03 a	0.62 ± 0.02 b	0.81 ± 0.03 b
	Ethanol	1.14 ± 0.01 b	0.08 ± 0.01 fg	0.00 ± 0.00 c
Stem	Aqueous	0.79 ± 0.03 de	0.30 ± 0.01 d	0.00 ± 0.00 c
	Ethanol	0.95 ± 0.01 c	0.17 ± 0.02 e	0.00 ± 0.00 c
Leaf	Aqueous	0.86 ± 0.02 d	0.46 ± 0.02 c	0.00 ± 0.00 c
	Ethanol	0.80 ± 0.01 de	0.07 ± 0.02 fg	0.00 ± 0.00 c
Flower	Aqueous	0.12 ± 0.02 f	0.11 ± 0.01 f	0.00 ± 0.00 c
	Ethanol	0.83 ± 0.02 de	0.06 ± 0.01 fg	0.00 ± 0.00 c
Pericarp	Aqueous	1.15 ± 0.03 b	0.85 ± 0.03 a	0.75 ± 0.04 b
	Ethanol	0.79 ± 0.03 de	0.11 ± 0.01 f	0.00 ± 0.00 c
Seed	Aqueous	0.94 ± 0.02 c	0.05 ± 0.01 g	0.96 ± 0.01 a
	Ethanol	0.78 ± 0.02 e	0.06 ± 0.02 fg	1.00 ± 0.09 a

^*1*^ The data represent the means ± SD of three determinations. Values with the same letter in one column are not significantly different (*p* = 0.01) from each other.

**Figure 3:**
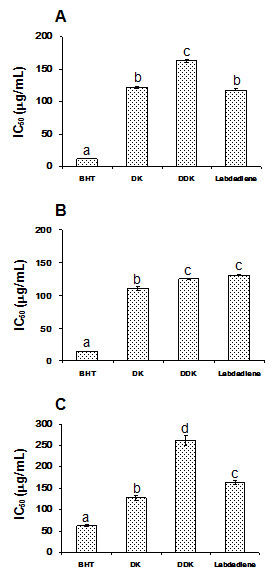
Effects of DK, DDK and labdadiene on DPPH (A), ABTS (B) and PMS-NADH (C) radical scavenging inhibitions.

The inhibitory properties of isolated compounds on antioxidant are shown in Figure 3. DK showed the highest inhibitory properties on DPPH, ABTS and PMS-NADH radical scavenging assays (IC_50_ = 122.14 ± 1.40, 110.08 ± 3.34and 127.78 ± 4.75 μg/ml, respectively). On statistical analysis of the result, isolated compounds were weaker inhibitors on antioxidant than BHT (*p* = 0.01).

**Figure 4:**
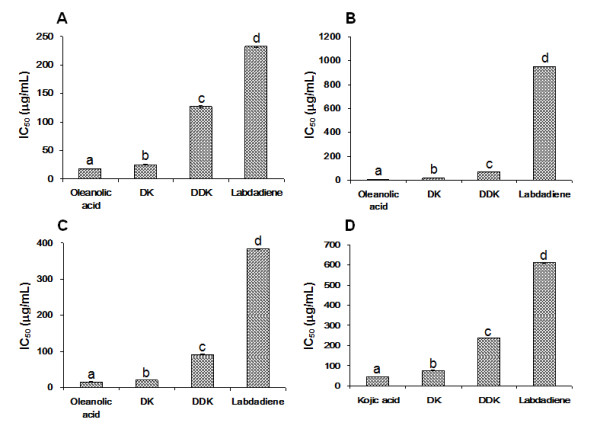
Inhibitory effects of DK, DDK and labdadiene on collagenase (A), elastase (B), hyaluronidase (C) and tyrosinase (D) activities.

Among the isolated compounds, DK showed stronger inhibitory activities against collagenase, elastase, hyaluronidase and tyrosinase (IC_50_ = 24.93 ± 0.97, 19.41 ± 0.61, 19.48 ± 0.24 and 76.67 ± 0.50 μg/ml, respectively) than DDK and labdadiene. However, isolated compounds had lower inhibitory effect than oleanolic acid [Figure 4] (*p* = 0.01).

## Discussion

Free radicals, such as ROS, are known to cause damage to cells during the process of aging thereby leading to a wide range of degenerative diseases [23]. Furthermore, the interactions between human body and the environment make the skin chronically exposed to both endogenous and environmental pro-oxidant agents which aid to the generation of ROS [24]. One of the important functions of skin is to protect from harmful environments. However, due to unusual disruption of connective tissues, the formation of free radicals, and the ultraviolet radiation result in skin wrinkling and pigmentation [25]. Several studies have explored on the prevention of these the skin abnormalities using plant extracts and phenolic compounds contribute in curing of skin diseases. Plant extracts such as pomegranate [26], tea [27] and wine [28] extracts have been shown to reduce the oxidative damage of UV light in skin. Purified phenolic compounds such as anthocyanins [29], proanthocyanidin [30] and EGCG [31] were found to inhibit the UV-radiation-induced oxidative stress and cell damage in human keratinocytes.

In this study, we screened the inhibitory activities of extracts obtained from six different structures of *A. zerumbet* with aqueous and ethanol extracts for ranking the efficacies. The results of preliminary screening showed that the rhizome and seed aqueous extracts had high antioxidant. Likewise, we found that the seed and rhizome aqueous extracts contained higher amounts of total phenolic contents, which may be the reasons for stronger radical scavenging activities. For skin diseases-related enzymes assays, rhizome aqueous extract exhibited the strongest activities in three of the performed assays while pericarp aqueous extract had better result in the collagenase inhibition. We quantified the amount of DK, DDK and labdadiene in all of test samples. The rhizome aqueous extract contained high amount of three compounds that may be the reason for stronger inhibitory properties. The inhibitory properties of these compounds have been published. For instance, DK had inhibitory effect on human platelet aggregation [32]. Beside, Upadhyay et al. [12] presented that DK strongly inhibited HIV-1 integrase and neuraminidase activities. Furthermore, Chompoo et al. [13] reported that labdadiene had strong reduction of advanced glycation end products formation. We further continued to study the potency of three isolated compounds isolated from the rhizomes on antioxidant and skin diseases-related enzymes activities.

Among the isolated compounds, DK exhibited the strongest activity in all of antioxidant assays. DK and DDK are the pyrone compounds, interestingly these compounds differ with one another by a single π-bond at C7-C8. DK contains a double bond and it seems that the presence of double bond has significant impact on antioxidant. Singlet oxygen reacts directly with the double bond of the unsaturated fatty acid by a concerted addition, the so-called “ene” reaction. This is a concerted reaction between singlet oxygen and carbon-carbon double bond in which the oxygen molecule is inserted at either carbon atom of the C = C bond [33,34]. Heim et al. [35] have found that flavonoids with a double bond are the better antioxidant. The double bond in the heterocycle or polymerization of the nuclear structure increases activity by affording a more stable flavonoid radical through conjugation and electron delocation. Moreover, Stahl et al. [36] reported that due to conjugated double bond in the polyene backbone of carotenoid structure, a major role in the protection of plants against photooxidative processes, which determines their light absorbing properties and influences the antioxidant activity. They are also part of the antioxidant defense system in animals and humans.

The skin diseases-related enzyme assays revealed that DK also has strong activity in among of isolated compounds. Several previous studies have been reported the ability of pyrone to exhibit skin diseases-related enzymes. Hosoe et al. [37] reported that γ-pyrone derivative, lepidepyrone, was a major hyaluronidase inhibitory compound. Cook et al. [38] presented that 3-(1-oxoalkyl)-4-hydroxy-6-alkyl-2-pyrone was found to be most effective to inhibit human sputum elastase. Huang et al. [39] demonstrated that dimeric naphtha-γ-pyrone, rubrofusarin, exhibited moderate tyrosinase activity, in addition, 6-*n*-pentyl-α-pyrone have been recorded as a potent tyrosinase inhibitor [40]. Moreover, pyrone have been presented to restrain other diseases. Hong [41] reported that tricyclic analogs and tricyclic pyrone compounds directly bind to and inhibit formation of amyloid-β (Aβ) aggregates in cell model representing Alzheimer’s disease. Yan et al. [42] mentioned that 3-hydroxy-4-pyrones and 5-amino-3-hydroxy-4-pyrone derivatives have been evaluated as inhibitor of matrix metalloproteinases. Hence we assume that DK is a pyrone compound and contain carbon double bond which may have a substantial role in inhibiting the skin diseases-related enzymes.

## Conclusion

This study revealed that the rhizome aqueous extract of *A. zerumbet* had indicated strong antioxidant and skin diseases-related enzymes. Since isolated compounds from rhizome of *A. zerumbet*, DK, was identified as a moderated potent inhibitor of antioxidant. Also, it was indicated that there was strong inhibition of skin diseases-related enzymes. Our results showed that DK have probable applications in preventing the skin against photo-oxidation. However further researches are necessary to use it as a lead compounds in drug designing.

## Abbreviations

DPPH, 1,1-Diphenyl-2-picrylhydrazyl; BHT, tert-butyl hydroxytoluene kojic acid,gallic acid; ABTS, 2,2-azino-bis(3-ethylbenzothiazoline-6-sulfonic acid); NBT, Diammonium salt nitro blue tetrazolium; PMS, Phenazine methosulfate; BSA, Bovine serum albumin; NADH, Nicotinamide adenine dinucleotide-reduced; SANA, N-succ-(Ala)3-nitroanilide; FALGPA, N-[3-(2-furyl)acryloyl]-Leu-Gly-Pro-Ala.

## Competing interest

The authors declare that they have no competing interests.

## Authors’ contributions

J was responsible for the conception and design, carried out all experiments, performed data analysis and drafted the manuscript. A was responsible for the conception and drafted the manuscript. F participated in the laboratory investigation and drafted the manuscript. S made substantial contribution to conception and revised it critically for important intellectual content and corresponding author. All authors read and approved the final manuscript.

## Pre-publication history

The pre-publication history for this paper can be accessed here:

http://www.biomedcentral.com/1472-6882/12/106/prepub

## References

[B1] Ndiaye M Philippe C Mukhtar H Ahmad N The grape antioxidant resveratrol for skin disorders: promise, prospects, and challenges Arch Biochem BiophysI 2011 508 164 170 10.1016/j.abb.2010.12.030 PMC3060966 21215251

[B2] Svobodová A Psotová J Walterová D Natural phenolics in the prevention of UV-induced skin damage A review. Biomed Papers 2003 147 137 145 15037894

[B3] Bickers DR Athar M Oxidative stress in the pathogenesis of skin disease J Invest Dermatol 2006 126 2565 2575 10.1038/sj.jid.5700340 17108903

[B4] Niederländer HAG van Beek TA Bartasiute A Koleva II Antioxidant activity assays on-line with liquid chromatography J Chromatogr A 2008 1210 121 134 10.1016/j.chroma.2008.09.061 18849036

[B5] Thring TSA Hili P Naughton DP Anti-collagenase, anti-elastase and anti-oxidant activities of extracts from 21 plants BMC complement Altern Med 2009 9 27 10.1186/1472-6882-9-27 19653897 PMC2728709

[B6] Lee KK Kim JH Inhibitory effects of 150 plant extracts on elastase activity, and their anti-inflammatory effects Int J Cosmet Sci 1999 21 71 82 10.1046/j.1467-2494.1999.181638.x 18505532

[B7] Barla F Higashijima H Funai S Sugimoto K Harada N Yamaji R Fujita T Nakano Y Inui H Inhibitive effects of alkyl gallates on hyaluronidase and collagenase Biosci Biotechnol Biochem 2009 73 2335 2337 10.1271/bbb.90365 19809167

[B8] Tief K Hahne M Schmidt A Beermann F Tyrosinase, the key enzyme in melanin systhesis, is expressed in murine brain Eur J Biochem 1996 241 12 16 10.1111/j.1432-1033.1996.0012t.x 8898882

[B9] Murakami S Li W Matsuura M Satou T Hayashi S Koike K Composition and seasonal variation of essential oil in Alpinia zerumbet from Okinawa Island J Nat Med 2009 63 204 208 10.1007/s11418-008-0306-4 19067113

[B10] De Araújo Pinho FVS Coelho-de-Souza AN Morais SM Santos CF Leal-Cardoso JH Antinociceptive effect of the essential oil of Alpinia zerumbet on mice Phytomedicine 2005 12 482 486 10.1016/j.phymed.2004.04.006 16008125

[B11] De Moura RS Emiliano AF de Carvalho LCRM Souza MAV Guedes DC Tano T Resende AC Antihypertensive and endothelium-dependent vasodilator effects of Alpinia zerumbet, a medicinal plant J Cariovasc Pharmacol 2005 46 288 294 10.1097/01.fjc.0000175239.26326.47 16116333

[B12] Elzaawely AA Xuan TD Tawata S Essential oil, kava pyrones and phenolic compounds from leaves and rhizomes of Alpinia zerumbet (Pers.) B.L. Burtt. & R.M. Sm. And their antioxidant activity Food Chem 2007 103 486 494 10.1016/j.foodchem.2006.08.025

[B13] Upadhyay A Chompoo J Kishimoto W Makise T Tawata S HIV-1 integrase and neuraminidase inhibitors from Alpinia zerumbet J Agric Food Chem 2011 59 2857 2862 10.1021/jf104813k 21306110

[B14] Chompoo J Upadhyay A Kishimoto W Makise T Tawata S Advanced glycation end products inhibitors from Alpinia zerumbet rhizomes Food Chem 2011 129 709 715 10.1016/j.foodchem.2011.04.034 25212289

[B15] Boskou G Salta FN Chrysostomou S Mylona A Chiou A Andrikopoulos NK Antioxidant capacity and phenolic profile of table olives from the Greek market Food Chem 2006 94 558 564 10.1016/j.foodchem.2004.12.005

[B16] Hsu CF Peng H Basle C Travas-Sejdic J Kilmartin PA ABTS·+ scavenging activity of polypyrrole, polyaniline and poly (3,4-ethylenedioxythiophene) Polym Int 2011 60 69 77 10.1002/pi.2912

[B17] Lau KM He ZD Dong H Fung KP But PPH Anti-oxidative, anti-inflammatory and hepato-protective effects of Ligustrum robustum J Ethnopharmacol 2002 83 63 71 10.1016/S0378-8741(02)00192-7 12413708

[B18] Djeridane A Youffi M Nadjemi B Boutassouna D Stocker P Vidal N Antioxidant activity of some algerian medicinal plants extracts containing phenolic compounds Food Chem 2006 97 654 660 10.1016/j.foodchem.2005.04.028

[B19] Van Wart HE Steinbrink DR A continuous spectrophotometric assay for Clostridium histolyticum collagenase Analyt Biochem 1981 113 356 365 10.1016/0003-2697(81)90089-0 6269461

[B20] Kraunsoe JAE Claridge TDW Lowe G Inhibition of human leukocyte and porcine pancreatic elastase by homologues of bovine pancreatic trypsin inhibitor Biochemistry 1996 35 9090 9096 10.1021/bi953013b 8703913

[B21] Kim JH Byun JC Bandi AKR Hyun CG Lee NH Compounds with elastase inhibition and free radical scavenging activities from Callistemon lanceolatus J Med Plant Res 2009 3 914 920

[B22] Tadtong S Viriyaroj A Vorarat S Nimkultat S Suksamrarn S Antityrosinase and antibacterial activities of mangosteen pericarp extract J Health Res 2009 23 99 102

[B23] Duan XJ Zhang WW Li XM Wang BG Evaluation of antioxidant property of extract and fractions obtained from a red alga, Polysiphonia urceolata Food Chem 2006 95 37 43 10.1016/j.foodchem.2004.12.015

[B24] Briganti S Picardo M Antioxidant activity, lipid peroxidation and skin disease What’s new. J Eur Acad Dermatol Venereol 2003 17 663 669 10.1046/j.1468-3083.2003.00751.x 14761133

[B25] Kim HH Lee MJ Lee SR Kim KH Cho KH Eun HC Chung JH Augmentation of UV-induced skin wrinkling by infrared irradiation in hairless mice Mech Ageing Dev 2005 126 1170 1177 10.1016/j.mad.2005.06.003 16118013

[B26] Afaq F Zaid MA Khan N Dreher M Mukhtar H Protective effect of pomegranate-derived products on UVB-mediated damage in human reconstituted skin Exp Dermatol 2009 18 553 561 10.1111/j.1600-0625.2008.00829.x 19320737 PMC3004287

[B27] Wang ZY Huang MT Lou YR Xie JG Reuhl KR Newmark HL Ho CT Yang CS Conney AH Inhibitory effect of black tea, green tea, decaffeinated black tea, and decaffeinated green tea on Ultraviolet B Light-induced skin carcinogenesis in 7,12-dimethylbenz[a]anthracene-initiated Cancer Res 1994 54 3428 3435 8012962

[B28] Matito C Agell N Sanchez-Tena S Torres JL Cascante M Protective effect of structurally diverse grape procyanidin fractions against UV-induced cell damage and death J Agric Food Chem 2011 59 4489 4495 10.1021/jf103692a 21405100

[B29] Tsoyi K Park HB Kim YM Chung JI Shin SC Shim HJ Lee WS Seo HG Lee JH Chang KC Kim HJ Protective effect of anthocyanins from black soybean seed coats on UVB-induced apoptotic cell death in vitro and in vivo J Agric Food Chem 2008 56 10600 10605 10.1021/jf802112c 18959412

[B30] Mantena SK Katiyar SK Grape seed proanthocyanidins inhibit UV-radiation-induced oxidative stress and activation of MAPK and NK-κB signaling in human epidermal keratinocytes Free Radical Biol Med 2006 40 1603 1614 10.1016/j.freeradbiomed.2005.12.032 16632120

[B31] Tobi SE Gilbert M Paul N McMillan TJ The green tea polyphenol, epigallocatechin-3-gallate, protects against the oxidative cellular and genotoxic damage of UVA radiation Int J Cancer 2002 102 439 444 10.1002/ijc.10730 12432544

[B32] Jantan I Raweh SM Sirat HM Jamil S Yasin YHM Jalil J Jamal JA Inhibitory effect of compounds from Zingiberaceae species on human platelet aggregation Phytomedicine 2008 15 306 309 10.1016/j.phymed.2007.08.002 17913483

[B33] Chan HWS Photo-sensitized oxidation of unsaturated fatty acid methyl esters The identification of different pathways. JAOCS 1977 54 100 104

[B34] Terao J Matsushita S The isomeric compositions of hydroperoxides produced by oxidation of arachidonic acid with singlet oxygen Agric Biol Chem 1981 45 587 593 10.1271/bbb1961.45.587

[B35] Heim KE Tagliaferro AR Bobilya DJ Flavonoid antioxidants: chemistry, metabolism and structure-activity relationships J Nutr Biochem 2002 13 572 584 10.1016/S0955-2863(02)00208-5 12550068

[B36] Stahl W Sies H Antioxidant activity of carotenoids Mol Aspects Med 2003 24 345 351 10.1016/S0098-2997(03)00030-X 14585305

[B37] Hosoe T Sakai H Ichikawa M Itabashi T Ishizaki T Kawai K Lepidepyrone, a new γ-pyrone derivative, from Neolentinus lepideus, inhibits hyaluronidase J Antibiot 2007 60 388 390 10.1038/ja.2007.53 17617697

[B38] Cook L Ternai B Ghosh P Inhibition of human sputum elastase by substituted 2-pyrone J Med Chem 1987 30 1017 1023 10.1021/jm00389a010 3647139

[B39] Huang HB Feng XJ Liu L Chen B Lu YJ Ma L She ZG Lin YC Three dimeric naptho-γ-pyrones from the mangrove endophytic fungus Aspergillus tubingensis isolated from Pongamia pinnata Planta Med 2010 76 1888 1891 10.1055/s-0030-1249955 20506081

[B40] Li X Kim MK Lee U Kim SK Kang JS Choi HD Son BW Myrothenones A and B, cyclopentenone derivatives with tyrosinase inhibitory activity from the marine-direved fungus Myrothecium sp Chem Pharm Bull 2005 53 453 455 10.1248/cpb.53.453 15802853

[B41] Hong HS Rana S Barrign L Shi A Zhang Y Zhou F Jin LW Hua DH Inhibition of Alzheimer’s amyloid toxicity with a tricyclic pyrone molecule in vitro and in vivo J Neurochem 2009 108 1097 1108 10.1111/j.1471-4159.2008.05866.x 19141069 PMC2748761

[B42] Yan YL Cohen SM An efficient synthesis of 5-amido-3-hydroxy-4-pyrones as inhibitors of matrix metalloproteinases Org Lett 2007 9 2517 2520 10.1021/ol0707665 17521196 PMC2531216

